# Photometric method for dual targeting of surface and surface-associated proteins on extracellular vesicles in the multiparametric test

**DOI:** 10.3389/fmolb.2022.917487

**Published:** 2022-10-25

**Authors:** Lee-Ann Marie Clegg, Jenni Kathrine Sloth, Rikke Bæk, Malene Møller Jørgensen

**Affiliations:** ^1^ Department of Clinical Immunology, Aalborg University Hospital, Aalborg, Denmark; ^2^ Department of Clinical Medicine, Aalborg University, Aalborg, Denmark

**Keywords:** extracellular vesicles, EV Array, multiparametric test, dual targeting, surface proteins

## Abstract

Extracellular vesicles (EVs) have become a topic of interest within the field of diagnostic biomarkers; however, recent developments in the study of EVs have increased the need for simpler but still comprehensive methods for characterization. Here, we describe how to simultaneously measure several surface or surface-associated proteins on EVs using a multiparametric microarray-based analysis termed Extracellular Vesicle Array (EV Array), which is developed to catch and phenotypically characterize small EVs. Previously, this analysis has been limited to measuring only one fluorescent signal per analysis. The analysis relies on antibodies printed onto a solid surface, for catching the EVs carrying the specific surface or surface-associated proteins, and on the subsequent fluorescent detection. For the optimization of detection, two antibodies with attached Cy3 or Cy5 were added to various combinations of the EV surface or surface-associated proteins: CD9, CD63, CD81, flotillin-1, and HSP90. In this study, the EV surface or surface-associated proteins were analyzed in human plasma from six healthy subjects. Changes observed in signal intensities from Cy3 and Cy5 related specifically to these combinations and allowed for a comparison of the two different fluorescent signals. When comparing the results, it was observed that it is possible to measure the EV surface or surface-associated proteins at both 532 nm (Cy3) and 635 nm (Cy5) simultaneously without a significant change in signals from the detection molecules. This allows us to measure multiple EV marker proteins in a single analysis, thereby more quickly finding complex biomarker patterns in a sample.

## 1 Introduction

Extracellular vesicles (EVs) have become an increasingly studied topic over the last decade as potential biomarkers in medical diagnostics. EVs are nanosized molecules that are highly abundant in most biofluids, and they play a vital role in regulating several biological functions by mediating cellular communication (Serrano-Pertierra et al., 2020). EVs are therefore promising candidates for the early detection of diseases, monitoring treatments, and as prognosis indicators, among others (Revenfeld et al., 2014; Schou et al., 2020). The EVs are released by cells and range from several nanometers to a few micrometers and are enclosed by a bi-layered plasma membrane. They are often classified by their composition, biogenetic pathway, and physical characteristics and contain bio-macromolecules, which they transport through biofluids over a long range inside the body (Kowal et al., 2016; Zendrini et al., 2020).

No proteins are found to be constitutively associated with the membrane of EVs; however, several markers have been identified to be more abundantly present and are often considered general EV markers. The tetraspanins CD9, CD63, and CD81 are some of the most abundant proteins on the EV surface, whereas the heat shock proteins (HSPs) and lipid rafts like flotillin-1, among others, are generally associated with the EV lumen or the EV corona (Chaput and Théry 2011; Mir and Goettsch 2020; Tóth et al., 2021).

Several methods for EV protein characterization exist; however, it is challenging to choose the most optimal one (Ramirez et al., 2018). The main issue is that many methods are limited by sample purification, labeling, and selection of optimal combinations of biomarkers. Generally accepted methods for characterization include antibody-based protein microarrays. The protein microarray is a well-known technique in which various sample types can be applied in the search for antibodies or antigens. Advantages such as parallel measurement of several proteins, fast analysis, high sensitivity, cost-effective benefits, and no need for EV purification are some of the factors that make this technique superior when compared with other methods.

In 2013, an extended protein microarray termed the EV Array was presented. The EV Array offers detection and phenotypic characterization of small EVs (sEVs) in a high-throughput approach with a low sample volume (≥10 µl) (Jørgensen et al., 2013). This technique relies on antibodies printed onto a solid surface, to catch the targeted EV surface or surface-associated proteins, and on the subsequent addition of fluorescent detection molecules for sEV profiling. A cocktail of selected biotinylated antibodies targeting the EV markers CD9, CD63, and CD81 is normally used for detection and profiling (Jørgensen et al., 2015, 2013).

Several studies using the EV Array have shown stable detection of EV proteins by the use of fluorescent-labeled streptavidin together with biotinylated antibodies, which accumulate one signal for each printed target (Jørgensen et al., 2015, 2021). This study demonstrates a new approach for the detection of surface or surface-associated proteins in the EV Array method where two detection molecules are applied for the identification of individual proteins, resulting in two signals for each printed target, one from each detected target. For this demonstration, CD9, CD63, CD81, HSP90, or flotillin-1 are targeted since they are all found in EVs (Chaput and Théry 2011; Mir and Goettsch 2020). Since EVs have great potential as diagnostic biomarkers, this method could be useful when searching for multiple targets on the surface of sEVs and furthermore avoid performing different EV analyses for the same sample, thereby reducing time, use of valuable samples, and cost of EV analysis.

## 2 Materials and methods

### 2.1 EV Array

#### 2.1.1 Preparation of the EV Array

**FIGURE 1:**
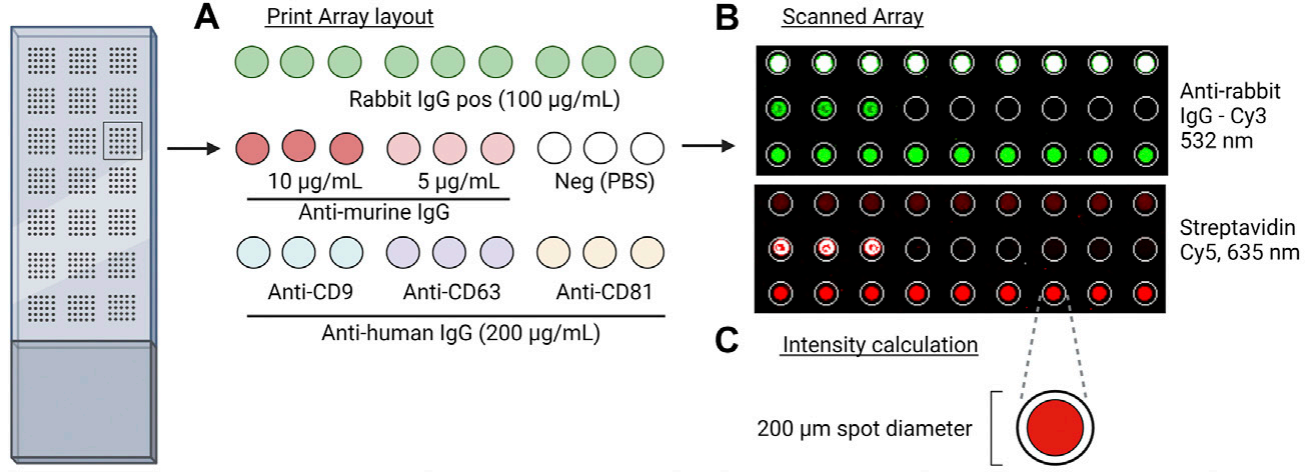
Overview of the EV Array method. **(A)** Print layout of antibodies (murine anti-human IgG), positive control (biotin-labeled anti-murine IgG and rabbit IgG), and negative control (PBS) for the analysis. Slides were placed into a 96-multi-well cassette, and 10 µL of the sample was added and incubated overnight. After the addition of detection antibodies and fluorescent molecules, the slides were scanned. **(B)** Scanned images at 635 nm and 532 nm of controls and captured EVs by the murine and rabbit antibodies, respectively. **(C)** Fixed spot area with a diameter of 200 µm applied for quantitative analysis.

The antibody array was prepared on epoxysilane-coated slides (75.6 × 25 mm, Schott MINIFAB, Germany) using the microarray printer sciFLEXARRAYER S12 using a size 60 piezo capillary with type 3 coating (Scienion AG, Berlin, Germany). A temperature range between 18 and 20°C and a humidity level between 55% and 65% were maintained during the printing procedure. An array layout was prepared (Figure 1A) and used to print three antibodies against human CD9, CD81 (Ancell Corporation, Stillwater, MN, United States), and CD63 (Bio-Rad Laboratories Inc., Hercules, CA, United States) with a final concentration of 200 μg/mL, two positive control solutions with either biotinylated goat anti-mouse IgG (Novus Biologicals, Centennial, CO, United States) with a final concentration of 5 and 10 μg/mL or rabbit anti-*Mycoplasma pneumoniae* IgG (Abcam plc., Cambridge, United Kingdom) with a concentration of 100 μg/mL, and a negative control with phosphate-buffered saline (PBS). All solutions were prepared in a spotting buffer with a final concentration of 50 mM trehalose in PBS to ensure uniformity in drop size and to prevent evaporation. The slides were left to dry in the dark at room temperature (RT) until further analysis.

#### 2.1.2 Sample preparation

Venous peripheral blood was collected in CPDA tubes (Vacuette^®^, Greiner Bio-One, Kremsmünster, Austria) from six healthy human subjects at Aalborg University Hospital (North Region, Aalborg, Denmark) after approval from the local ethics legislation. The studies involving human participants were reviewed and approved by the Scientific Ethical Committee of Central Denmark and the Danish Data Protection Agency (2007-58-0015). Verbal consent was obtained from each individual stating the use of their blood for research purposes. Before plasma isolation, each blood sample was incubated for 1 h at RT prior to centrifugation at 1,800 *g* for 6 min, and the isolated plasma was stored at −40°C until further analysis.

#### 2.1.3 The EV Array procedure—catching and analysis of EVs

As a first step in the EV Array procedure, slides were placed in a high-throughput wash container (Arrayit Corporation, Sunnyvale, CA, United States), and a peristaltic pump was used to slowly add the blocking buffer (50 mM ethanolamine, 100 mM Tris, and 0.1% sodium dodecyl sulfate (pH 9)) into the container while stirring at 120 RPM. After 1 h incubation, the blocking buffer was replaced with wash buffer (0.05% Tween20^®^ in PBS), and stirring was changed to 200 RPM for 15 min at RT.

Before sample application, the slides were placed into a multi-well hybridization cassette (Arrayit Corporation, Sunnyvale, CA, United States). A total volume of 100 µL sample was then added to each well, diluted to 1:10 with incubation buffer (0.5X casein blocking buffer (Sigma-Aldrich, MO, United States) + 0.1% Tween20^®^ in PBS), and incubated on an orbital shaker at 450 RPM for 2 h. Afterward, the cassettes were incubated at 4°C overnight.

After the incubation, the slides were removed from the cassette and washed for 15 min with wash buffer.

Five different combinations of detection antibodies were then applied, with one combination per slide. These antibodies and their combinations are listed in Table 1. The detection molecules applied were a mixture of biotinylated anti-human-CD9, -CD63, and -CD81 detection antibodies, referred to as a “cocktail” (Ancell Corporation, Stillwater, MN, United States), anti-human flotillin-1 and anti-human HSP90 (Abcam plc., Cambridge, UK), Cy5-labeled streptavidin (Life Technologies, Carlsbad, CA, United States), and Cy3-labeled goat anti-rabbit IgG (Millipore, Burlington, MA, United States).

**TABLE 1:** Five different combinations of detection antibodies and fluorescent molecules for visualization in the EV Array procedure.

Combination	Detection antibody	Fluorescent detection molecule
I	Anti-human flotillin-1	Cy3-labeled goat anti-rabbit IgG
II	Anti-human HSP90	Cy3-labeled goat anti-rabbit IgG
III	Cocktail*	Cy5-labeled streptavidin
IV	Cocktail* + anti-human flotillin-1	Cy5-labeled streptavidin + Cy3-labeled goat anti-rabbit IgG
V	Cocktail* + anti-human HSP90	Cy5-labeled streptavidin + Cy3-labeled goat anti-rabbit IgG

*Mix of biotinylated anti-human-CD9, -CD63, and -CD81 is referred to as a “cocktail.”

All antibodies were diluted to 1:1,500 in incubation buffer and incubated at RT for 2 h at 450 RPM. Following a 15 min wash procedure in wash buffer, the slides were incubated for 1 h with either Cy5-labeled streptavidin and/or Cy3-labeled goat anti-rabbit IgG diluted to 1:1,500 and 1:3,000, respectively (Table 1) (Jørgensen et al., 2015, 2013).

As the final step, all slides were washed for 15 min at RT with wash buffer and afterward with MilliQ water for 15 min at 200 RPM.

All slides were dried on a microarray high-speed centrifuge (Arrayit Corporation, Sunnyvale, CA, United States) and sequentially scanned using the InnoScan 710AL microarray scanner (Innopsys Inc., France) at 635 and 532 nm (Figure 1B).

#### 2.1.4 Data analysis

All microarray data were obtained using Mapix software version 9.1.0 (Innopsys Inc., France), and the total signal intensity from each spot, with a fixed size of Ø200 µm, was subtracted from the total signal intensity from the negative control (PBS) (Figure 1C). *p*-values were calculated using a Wilcoxon matched-pairs signed rank test, and *p*-values < 0.05 were considered significant.

Microsoft Excel 365 (Redmond, WA, United States) and GraphPad Prism version 8.0 (GraphPad Software, San Diego, CA, United States) were used to make the calculations, statistics, graphs, and heatmaps.

### 2.2 Western blot

#### 2.2.1 Isolating EVs

PBS was added to the plasma samples in the ratio of 1:1 and centrifuged at 13,200 g for 22 min at 4°C. Then, the supernatant from each sample was collected, filtered (0.22 µm), and ultracentrifuged (Avanti J-30I, rotor JA-30.50, Beckman Coulter, Brea, CA, United States) for 16 h at 100,000 g at 4°C. The supernatant was discarded, and PBS was applied to wash the pellet before ultracentrifugation at 100,000 g for 3 h at 4°C. After removal of the supernatant, the pellet was dissolved in 50 µL of Pierce™ RIPA buffer (Thermo Scientific, Rockford, United States) and stored at −40°C until further analysis.

#### 2.2.2 SDS-PAGE

The samples were mixed in the ratio of 3:1 with NuPAGE™ LDS sample buffer (4X) (Life Technologies™, Carlsbad, CA, United States). All solutions were then heated at 70°C for 10 min before being added to a precast NuPAGE™ Bis–Tris Gel 10% (Invitrogen, Carlsbad, CA, United States) in the XCell SureLock™ Mini-Cell electrophoresis system (Life Technologies™, Carlsbad, CA, United States). The system was prepared with 1X NuPAGE™ MES SDS Running Buffer (20X) (Life Technologies™, Carlsbad, CA, United States) and 10–250 kDa Precision Plus Protein™ WesternC™ standards (Bio-Rad, Hercules, CA, United States). Electrophoresis was then performed at 100 V for 10 min and subsequently at 140 V for 65 min, and finally, the gel was rinsed with MilliQ water before blotting.

#### 2.2.3 Immunoblotting and imaging

Blotting of the SDS gel was performed using an iBlot^®^2 Dry Blotting System (Thermo Scientific, Waltham, MA, United States) by applying the iBlot^®^2 NC Regular Stacks with a nitrocellulose membrane. Afterward, the membrane was placed in MilliQ water at RT for storage.

The membrane was incubated for 2.5 h at RT with specific primary and secondary antibodies in the iBind™ Western Device by applying the iBind™ Western System (Life Technologies™, Carlsbad, CA, United States). The procedure was performed according to the manufacturer’s protocol. Both the primary antibodies, anti-CD9 and anti-CD81 (Ancell Corporation, Stillwater, MN, United States), and the secondary antibody, HRP goat anti-mouse IgG (LI-COR^®^, NE, United States), added to 0.5 µL of Precision Protein StrepTactin-HRP conjugate (Bio-Rad, Hercules, CA, United States), were diluted to a final concentration of 1 μg/mL in iBind™ solution. As a final step, the blot was rinsed with MilliQ water before immunodetection was performed.

SuperSignal™ West Pico PLUS Chemiluminescent Substrate (Thermo Scientific, Rockford, United States) was applied for immunodetection where stable peroxide and luminol/enhancer solution were mixed in the ratio of 1:1 and incubated with the membrane for 5 min.

The membrane was imaged for 12 min at a maximum intensity using a C-DiGit Chemiluminescence Western Blot Scanner (LI-COR^®^, NE, United States) and visualized using Image Studio Digits software version 5.2 (LI-COR^®^, NE, United States).

## 3 Results and discussion

Epoxy-coated slides were chosen as the basis for the antibody microarray used for the capture of sEVs, and detection was performed using a high-resolution (16 bit and 5 µm pixel size) laser-based confocal scanner. Small EVs in unpurified samples were phenotypically analyzed using the EV Array, and purified sEVs were validated with Western blot (WB). This study focuses on the detection and separate measurements of multiple targets on sEVs and is presented as an optimization of the established EV Array method (Jørgensen et al., 2013). Therefore, sEV characteristics were not thoroughly investigated as recommended by the MISEV guidelines (Théry et al., 2018).

To capture sEVs, anti-CD9, -CD63, and -CD81 were printed in triplicate onto the slides. These antibodies were chosen since they are enriched in the EV membrane and are often considered general EV biomarkers (Kowal et al., 2016). The lipid raft protein, flotillin-1, and heat shock protein, HSP90, were used as EV targets along with CD9, CD63, and CD81 in an attempt to detect and separately measure multiple proteins on captured sEVs. The presence of all the EV proteins mentioned has been confirmed by other studies (Chaput and Théry 2011; Andreu and Yáñez-Mó 2014; Mir and Goettsch 2020).

**FIGURE 2:**
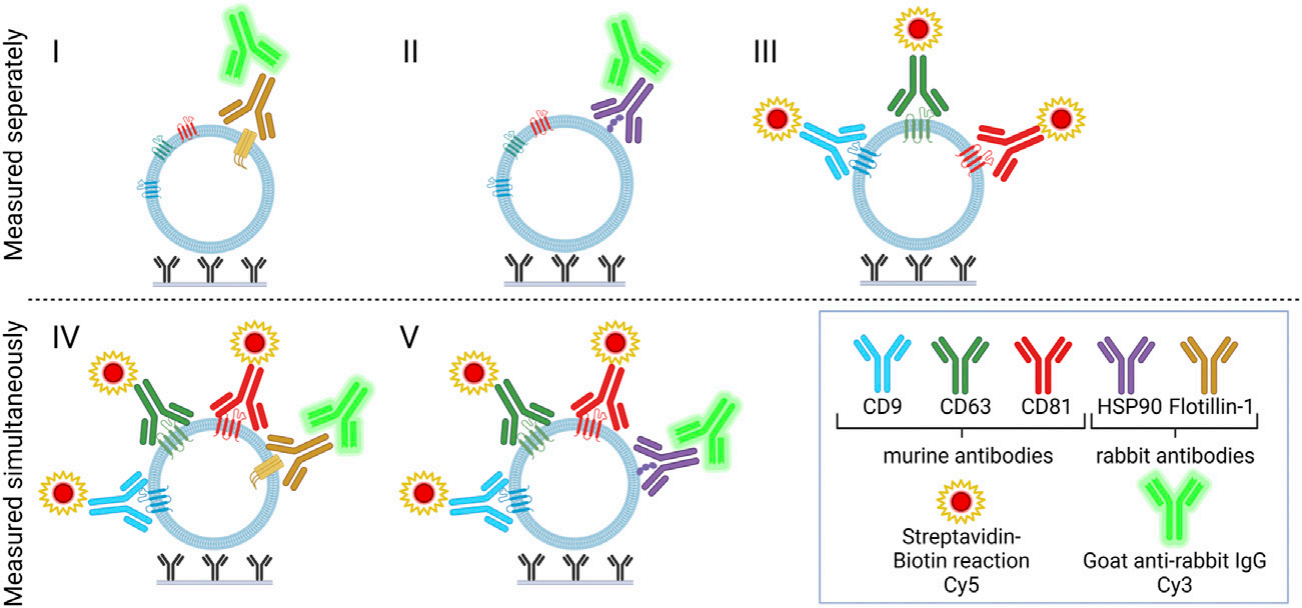
Immune-mediated EV capture and detection for dual targeting of surface or surface-associated proteins. Capture antibodies (murine), positive control (biotinylated IgG or rabbit anti-*Mycoplasma pneumoniae* IgG), and negative control (PBS) were printed onto glass slides. Slides were added to plasma samples and incubated overnight. For the detection of EVs, detection molecules (biotinylated murine anti-CD9, -CD63, and -CD81 and/or rabbit anti-HSP90 and -flotillin-1) were added, and two separate fluorescent signals were obtained with Cy5-labeled streptavidin and/or Cy3-labeled goat anti-rabbit IgG. Compositions I, II, and III were measured separately, whereas IV and V were measured simultaneously.

Two approaches for detection were combined to measure signals from Cy3- and Cy5-labeled molecules, respectively. Rabbit antibodies and biotinylated murine anti-human antibodies were applied to separate the two signals since Cy5-labeled streptavidin interacts with the biotinylated antibodies and the anti-rabbit IgG has an affinity for the rabbit antibodies. The five different combinations of detection antibodies are described in Table 1 and are illustrated in Figure 2.

### 3.1 EV Array analysis

The advantage of optimizing the EV Array method is that signals from more antibodies can be measured and evaluated simultaneously, which can be very useful to detect co-localization of antigens, which could be useful in a clinical setting. To illustrate the detection of multiple targets, five different compositions of detection antibodies were analyzed against plasma samples from six healthy blood donors (Table 1).

**FIGURE 3:**
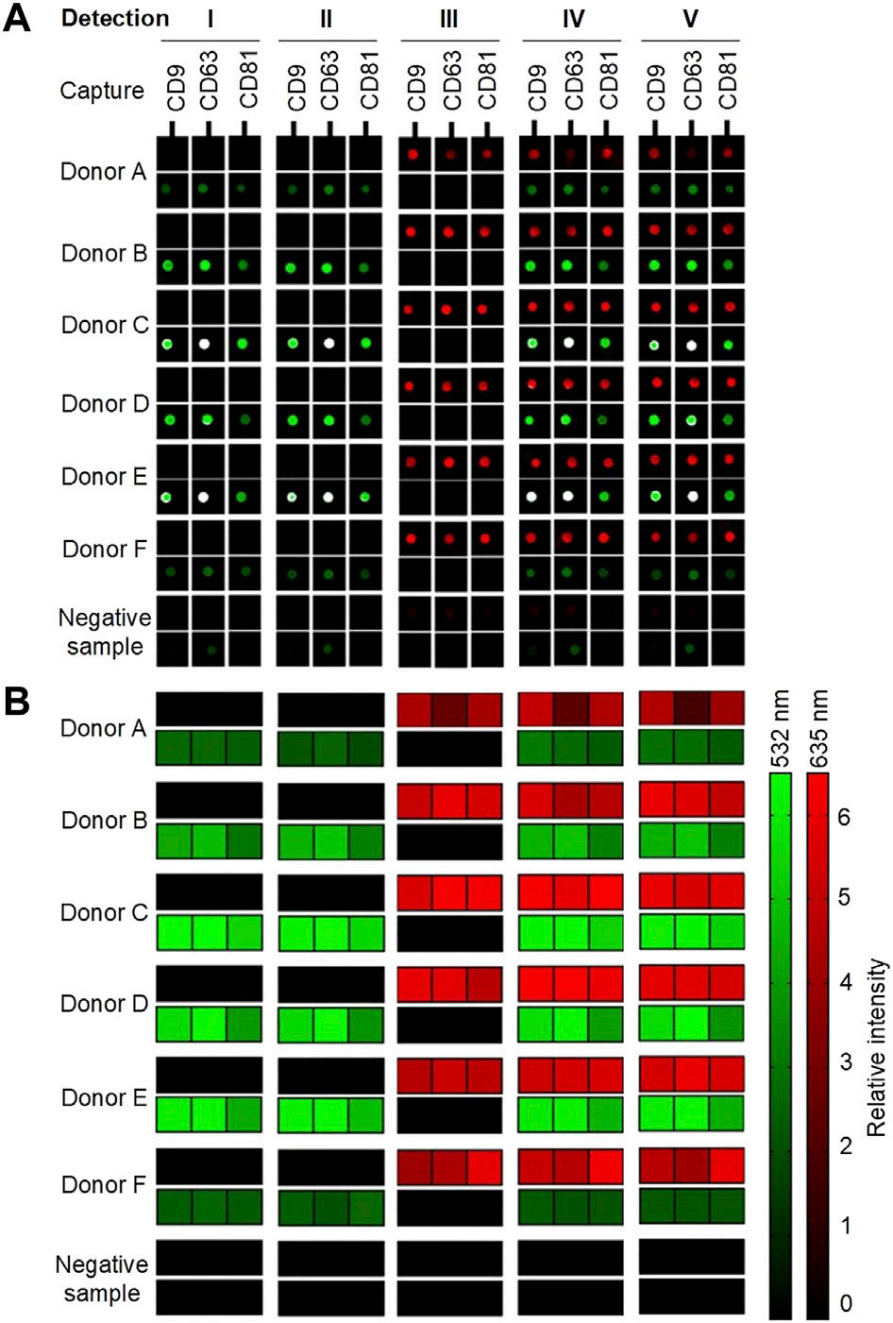
Overview of the results from the EV Array analysis of six donors **(A–F)** and a negative sample (incubation buffer). Five different compositions of detection antibodies were examined (Table 1; Figure 2). A cocktail with biotinylated murine anti-CD9, -CD63, and -CD81, rabbit anti-flotillin-1, and/or anti-HSP90 were analyzed separately and simultaneously. **(A)** Spots indicate the capture of sEVs in a sample with printed antibodies: CD9, CD63, and CD81. Red signals (635 nm) originate from Cy5-labeled streptavidin reacting with the biotinylated murine antibodies. Green signals (532 nm) originate from Cy3-labeled goat anti-rabbit IgG bound to the flotillin-1 and HSP90 rabbit antibodies. **(B)** Combined heatmap of the spot relative intensities (signal-to-background ratios). The red and green heatmaps summarize the intensities of the bound murine and rabbit antibodies, respectively.

For visualization, all samples with biotinylated antibodies were added to Cy5-labeled streptavidin, creating a red signal (635 nm). All samples with either anti-flotillin-1 or anti-HSP90 were, furthermore, added to a Cy3-labeled secondary goat anti-rabbit IgG antibody, resulting in a green signal (532 nm). A sample, with only incubation buffer, was used as a negative control sample to account for any secondary or unspecific contribution to the signal obtained by background fluorescence from either the plasma samples or printed capture antibodies. The results are presented in Figures 3A,B with either scanned images of represented spots or a heatmap of the calculated relative intensity of each detected surface-associated target protein.

As shown in Figures 3A,B, it was possible in all donor samples to get two separate signals simultaneously from the two detection molecules: Cy5-labeled streptavidin and Cy3-labeled goat anti-rabbit IgG in each of the five tested conditions. Small EVs were captured in all samples, and the EV markers CD9, CD63, and CD81 were, as expected, detected with a relatively high signal intensity with Cy5-labeled streptavidin. It is observed in donors A and F that the presence of flotillin-1 and HSP90 is quite low when compared to the other donors. This might be due to biological variance between individuals. In the negative sample, it can be observed, as shown in Figure 3A, that a small background signal is obtained in the Cy3 channel (532 nm). A small investigation study was conducted (results not shown) which showed that unspecific binding occurs between the Cy3-labeled goat anti-rabbit IgG antibody and the printed murine anti-human CD9, CD63, and CD81 antibodies on the slide. To account for this contribution to the Cy3 signals, all results have been subtracted from the signal from the negative sample (with only incubation buffer added) before further analysis. Future research could consider testing other fluorophores and detection antibodies in an attempt to remove all unspecific background signals.

**FIGURE 4:**
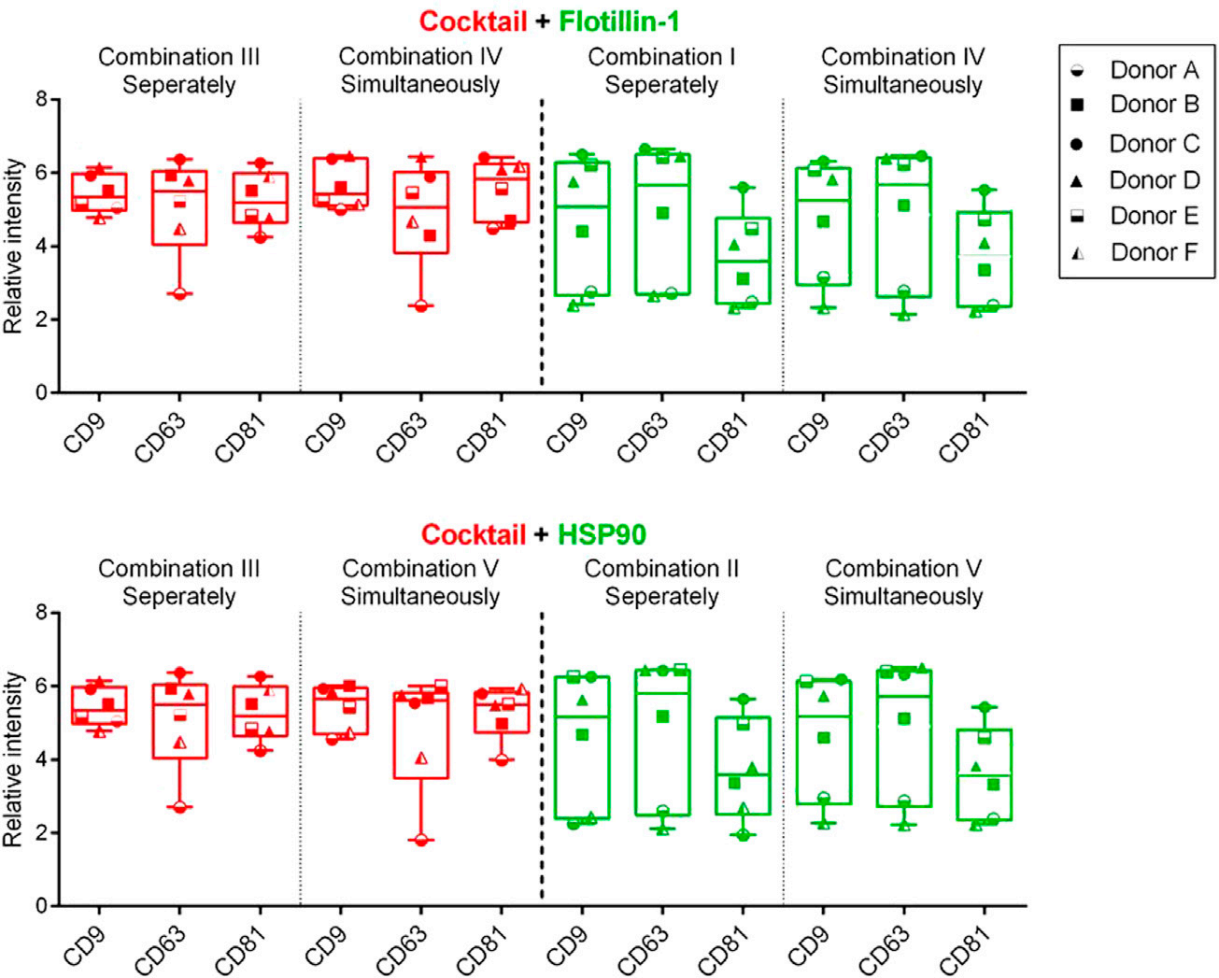
Box and whisker plot (min to max) of the background-corrected intensities obtained from spots with captured sEVs by CD9, CD63, and CD81 antibodies (Y-axis). Data include compiled results from six donors (A–F) and five different compositions (I–V) of detection antibodies. A cocktail of murine antibodies, CD9, CD63, and CD81, or rabbit antibodies, flotillin-1 and HSP90, was measured separately or simultaneously. Cy5-labeled streptavidin (red) and Cy3-labeled IgG (green) were applied for detection. *p*-values, between separate and simultaneous measurements, were calculated using the Wilcoxon matched-pairs signed rank test (all *p*-values > 0.05).

To test if it was possible to measure the individual biomarkers within the same relative signal intensity range, a univariate chart was used to compare the differences of the Cy3 (green) or Cy5 (red) signals between the measurements performed simultaneously and separately. This analysis was conducted by compiling the results obtained from each biomarker from all six donors (Figure 4).

Figure 4 shows that a similar level of relative intensity was obtained for all biomarkers in all compositions measured either separately or simultaneously. Based on these results, *p*-values furthermore show no significant difference between the detected signals in almost all of the tested antibody compositions (Tables 2, 3). These results indicate that it was possible to measure signals from Cy3- and Cy5-labeled molecules simultaneously on sEVs using the EV Array. When focusing on the sEVs captured by anti-CD9 and anti-CD63, a comparison of the Cy3 or Cy5 measurements of the two compositions (IV and V) with anti-CD9, -CD63, -CD81, and -flotillin-1 or anti-CD9, -CD63, -CD81, and -HSP90 showed no significant difference when measured simultaneously (*p* = 0.16 and *p* = 0.56 or *p* = 0.31 and *p* = 0.69) (Table 2). Comparing all the results, it can be observed that only rabbit anti-flotillin-1 and rabbit anti-HSP90 bound to sEVs captured by CD81 show a small decrease in relative signal intensity compared to the other biomarkers (*p* = 0.03) (Table 2). This decrease might suggest a lower number of CD81 tetraspanins associated with the sEV membrane for capture on the slides compared to CD9 and CD63 tetraspanins. As the distribution of the tetraspanins, among others, associated with the EV membrane varies and is affected by individual variance, it was expected that some biomarkers would not be detected with equal signal intensity. Previous studies have established the heterogeneity of EVs (Kowal et al., 2016). It should be noted that these results could be biased by the possibility of FRET pairing of Cy3 and Cy5. This could be avoided by applying other dyes such as Alexaflour instead of Cy5, as these fluorophores photobleach poorly and could decrease the possibility of FRET interference on the results (Snapp and Hegde 2006).

**TABLE 2:** *p*-values from simultaneously measured antibody compositions with Cy5- and Cy3-labeled molecules.

Capture antibody	Combination IV	Combination V	Significant difference
	Cocktail (Cy5)/flotillin-1 (Cy3)	Cocktail (Cy5)/HSP90 (Cy3)	
CD9	0.16	0.31	No
CD63	0.56	0.69	No
CD81	0.03	0.03	Yes

Relative signal intensities from a cocktail with anti-CD9, -CD63, and -CD81 are compared to either rabbit anti-flotillin-1 or rabbit anti-HSP90 relative signal intensities. *p*-values were calculated using the Wilcoxon matched-pairs signed rank test.

**TABLE 3:** *p*-values from the comparison of separately vs. simultaneously measured antibody compositions with Cy5- and Cy3-labeled molecules.

Capture antibody	Combination I	Combination II	Combination III	Significant difference
	Flotillin-1 (Cy3)	HSP90 (Cy3)	Cocktail (Cy5)	
CD9	0.84	0.84	>0.99	No
CD63	0.56	0.56	0.22	No
CD81	0.84	0.31	0.84	No

*p*-values were calculated using the Wilcoxon matched-pairs signed rank test.

### 3.2 Western blot analysis

**FIGURE 5:**
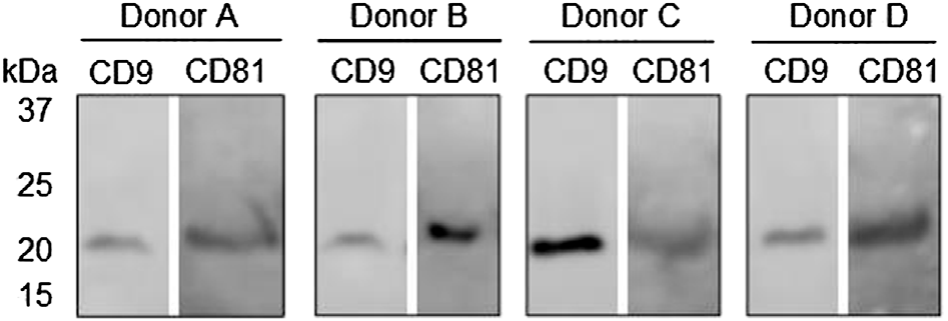
Western blot analysis of general EV biomarkers: CD9 and CD81 on purified EV samples from donors A, B, C, and D. Bands at approximately 24 kDa were expected with respect to both biomarkers.

Western blot analysis was applied to validate the presence of the general EV biomarkers, CD9 and CD81, in purified EV samples from donors A, B, C, and D. Figure 5 shows that there are bands between 21 and 25 kDa at both CD9 and CD81 in the tested samples from all four donors, which was as expected. These results indicate that EVs were present in the samples applied for the EV Array analysis. No comparison was made between the band intensities between the donors or the EV Array results due to differences in the purification procedure and sample material applied in each assay. To sum up, the results from the EV Array analysis clearly show that it was possible to target and measure two separate signals from antibodies with affinity for distinct proteins associated with the sEV surface. Today, not many methods allow for the simultaneous identification of multiple markers associated with the surface of EVs, which makes this technical advancement within EV research an attractive tool in future studies.

Phenotypical characterization of EVs using two or more fluorophores can, however, also be performed with flow cytometry. Even though this method is mostly used to measure microvesicles (100–1000 nm), other highly sensitive platforms like dedicated flow cytometry and advanced imaging flow cytometry have previously shown the ability to measure particles and synthetic nanospheres, consistent with sEVs, down to <30 nm and 20 nm, respectively (Ramirez et al., 2018; Botha et al., 2021). Although these platforms can detect molecules corresponding to sEVs, like the EV Array method, they lack high reproducibility, high throughput due to a need for several controls, increased sensitivity in regard to the optic settings, published benchmarks for detection of sEVs, and comparable data analysis strategies.

Another existing technology that uses two or more fluorophores for EV characterization is made by NanoView Biosciences that applies a standardized capture antibody selection and a limited number of detection antibodies of its own choice (Brighton, MA, United States). In comparison to both the flow cytometry methods and the technology made by NanoView Biosciences, the advantages of the EV Array method are: application of numerous specific capture biomarkers (>20 markers), customized selection of numerous detection antibodies, and a very small sample volume (≥10 µL). This allows for the detection of sEVs from specific cell types and tissues, among others, along with the application of more biomarkers of own choice, thereby creating a more user-friendly and customized assay platform.

## 4 Conclusion

This study represents the first time where two signals, measured at different wavelengths, have been applied in the EV Array method. We can conclude that it was possible to measure EV surface or surface-associated proteins at both 532 nm (Cy3) and 635 nm (Cy5) simultaneously without a significant change in signals from the detection molecules. Overall, this study provides an important analytical advantage within medical diagnostics since it allows for the simultaneous measurement of both general and specific biomarkers in a sample.

In the future, the detection of further biomarkers and their compatibility with the EV Array platform should be explored.

## Data Availability

The raw data supporting the conclusion of this article will be made available by the authors, without undue reservation.
